# Interferon-gamma Genetic Polymorphism and Expression in Kawasaki Disease

**DOI:** 10.1097/MD.0000000000003501

**Published:** 2016-04-29

**Authors:** Ying-Hsien Huang, Yu-Wen Hsu, Hsing-Fang Lu, Henry Sung-Ching Wong, Hong-Ren Yu, Hsing-Chun Kuo, Fu-Chen Huang, Wei-Chiao Chang, Ho-Chang Kuo

**Affiliations:** From the Department of Pediatrics and Kawasaki Disease Center, Kaohsiung Chang Gung Memorial Hospital and Chang Gung University College of Medicine, Kaohsiung, (Y-HH, H-RY, F-CH, H-CK); Department of Clinical Pharmacy, Taipei Medical University (Y-WH, H-FL, W-CC); The Ph.D. Program for Translational Medicine, College of Medical Science and Technology, Taipei Medical University and Academia Sinica (Y-WH); Department of Pharmacy, Taipei Medical University-Shuang Ho Hospital (H-FL, W-CC); Department of Clinical Pharmacy, College of Pharmacy, Taipei Medical University (HS-CW, W-CC); Master Program for Clinical Pharmacogenomics and Pharmacoproteomics, School of Pharmacy, Taipei Medical University, Taipei (W-CC, H-CK); and Institute of Nursing and Department of Nursing, Chang Gung University of Science and Technology, Kaohsiung, Taiwan (H-CK).

## Abstract

Kawasaki disease (KD) is a systemic vasculitis of unknown etiology. *IFNG* gene encoding interferon (IFN)-γ, produced by natural killer cells and T cells, has been suggested to play an important role in the immunopathogenesis of Kawasaki disease. The aim of this study was to examin the correlation of gene polymorphisms of the *IFNG* gene and plasma levels of IFN-γ in KD patients and their outcomes.

A total of 950 subjects (381 KD and 569 controls) were recruited. Three tagging single-nucleotide polymorphisms (rs2069718, rs1861493, rs2069705) were selected for TaqMan allelic discrimination assay. Clinical phenotypes, coronary artery lesions (CAL), coronary artery aneurysms (CAA) and intravenous immunoglobulin (IVIG) treatment outcomes were collected for analysis. Plasma IFN-γ levels were also measured with an enzyme-linked immunosorbent assay.

Polymorphisms of the *IFNG* gene were significantly different between the normal controls and KD patients. The G allele of rs1861493 conferred a better response to IVIG treatment in KD patients. AA allele frequencies of rs1861493 were also associated with a significantly higher risk of CAA in KD patients. Furthermore, the plasma IFN-γ level was lower in the AA allele than in the GG allele of rs1861493 both before and after IVIG treatment in KD patients.

This study provides the first evidence supporting an association between *IFNG* gene polymorphisms, susceptibility of KD, IVIG responsiveness, and plasma IFN-γ levels in KD patients.

## INTRODUCTION

Kawasaki disease (KD) is an acute febrile systemic vasculitis of unknown etiology, and it has a predilection for the involvement of coronary arteries in childhood.^1^ The clinical manifestations of KD are fever for >5 days, strawberry tongue, conjunctival inflammation, cervical lymphadenopathy, polymorphous rashes, and brawny edema of the hands and feet.^2^ In addition, KD is also the most common cause of acquired heart diseases in children.^2,3^ More than 20% of patients with KD may develop coronary artery lesions (CALs) if not given adequate treatment with intravenous immunoglobulin (IVIG), which in turn increase risk of coronary artery fistula formation,^4^ myocardial infarction, or coronary artery aneurysm (CAA).^5^ Clinical and epidemiology evidence indicates higher incidence rates of KD in Japan, followed by Korea and Taiwan, and lowest in Europe.^5^ Thus, susceptibility genetic polymorphisms might play an important role in the immunopathogenesis of KD.

The mounting evidence of the clinical and epidemiologic characteristics of KD strongly support the hypothesis that an infectious agent may be the inducing factor.^2^ Till now, most researchers believe that imbalance of the immune system and abnormal Th1/Th2/Treg profiles are the key immunopathogenesis in KD.^6–9^ IFN-γ, a Th1 cytokine secreted mostly by natural killer cells and CD8+ T cells, can recruit macrophages and T cells into coronary arteries, thereby contributing to the production of reactive oxygen species, stimulating matrix metalloproteinases, and inducing tissue factor expressions.^10,11^ Recently, it was shown that IFN-γ was significantly increased in KD patients before IVIG treatment and the higher level of IFN-γ was associated with patients with CAL than those without CAL.^12^ The *IFNG*, encoded IFN-γ, gene polymorphisms have been reported by various genetic association studies to also be implicated in asthma susceptibility,^13,14^ infectious disease,^15^ coronary artery disease^16^ and autoimmune diseases.^17,18^ Therefore, the aim of this study was to investigate in KD patients the correlation of *IFNG* gene polymorphisms and the plasma levels of IFN-γ and their outcomes.

## PATIENTS AND METHODS

### Patients

This study was approved by the Institutional Review Board of Chang Gung Memorial Hospital (IRB No. 98–0037B), and written informed consent was obtained from parents or guardians of the participants. Three hundred and eighty-one KD patients and 569 controls were studied. Blood samples from age-matched control subjects, who were admitted because of respiratory tract infections (such as acute pharyngitis, acute tonsillitis, croup, acute bronchitis, and acute bronchiolitis), were used for genotyping. All patients of KD were treated with a single high dose of IVIG (2 g/kg) in 12 hours, as previously described.^7^ Patients with symptoms that did not fully match the KD criteria of American Heart Association were excluded. We used SONOS 5500 or 7500 cardiac scanner (Philips, Andover, MA) with a 5- to 8-MHz sector phased array transducers in this study. All patients of KD underwent a series of pulse Doppler, two-dimensional, and color flow echocardiogram at least 3 times within 8 weeks from the onset of the illness as previously described.^7^ Echocardiographic follow-up was performed every 3 to 6 months in the first year for KD patients with abnormal coronary artery, and then once annually until the affected coronary arteries become normal, as in our previous studies.^7,19,20^ We used 2-dimensional echocardiography to visualize the diameter of the left and right coronary arteries on the parasternal short-axis view of the aorta.^4^ In an echocardiogram, a CAL is defined as the internal diameter of the coronary artery being >3 mm (4 mm, if the subject was older than 5 years) or the internal diameter of a segment being at least 1.5 times that of an adjacent segment.^21,22^ Transient coronary artery ectasia or dilatation, which disappeared within the initial 8 weeks after the onset of illness, was not identified as CAL.^21,22^ The CAA was defined as internal diameter of coronary artery being >4 mm or a segment being at least 1.5 times that of an adjacent segment in children older than 5 years according to JCS Joint Working Group.^21,22^ IVIG responsiveness (or resistance) was defined as defervescence 48 hours after the completion of IVIG administration and no fever (defined as a ear temperature >38°C) recurrence for at least 7 days, with marked normalization or improvement of inflammatory signs.^20,23^ Blood samples were immediately stored in heparin tubes, and were stored at −80°C until performing assay, as in our previous studies.^7^

### DNA Extraction

Blood cells were treated with 0.5% SDS lysis buffer and then protease K (1 mg/mL) for 4 hours at 60°C to digest the nuclear proteins. Total DNA was extracted using a Gentra extraction kit followed by 70% alcohol precipitation.

### Genotyping

In total, 3 tagging single-nucleotide polymorphisms (SNPs) of *IFNR* (rs2069718, rs1861493, rs2069705) with a minimum allelic frequency of >10% in the Han Chinese population were selected from the HapMap database (http://hapmap.ncbi.nlm.nih.gov/). The rs2069718 was located on the 5’UTR of *IFNG*, and rs1861493 and rs2069705 of the *IFNG* gene were located in the introns. Genotyping was carried out using a TaqMan Allelic Discrimination Assay (Applied Biosystems, Foster City, CA), as previously described.^24^ Quantification of PCR was performed using a 96-well microplate with an ABI 9700 Realtime PCR system, with the following thermal cycling conditions: denaturing at 95°C for 10 minutes, followed by 40 cycles of denaturing at 92°C for 15 seconds and annealing and extension at 60°C for 1 minute, as previously described.^24^ Fluorescence was quantified using System SDS software version 1.2.3 (Applied Biosystems).

### Measurement of Cytokines by an Enzyme-Linked Immunosorbent Assay

We used an enzyme-linked immunosorbent assay to measure human interferon (IFN)-γ according to the manufacturer's instructions.

### Statistical Analysis

All data are presented as the mean ± standard deviation. Quantitative data were analyzed using 1-way analysis of variance where appropriate. The least significant difference test was used for post-hoc testing where appropriate. Changes in the data before and after IVIG treatment were tested by the paired sample *t* test. All statistical analyses were performed by using SPSS version 13.0 for Windows XP (SPSS software, Inc, Chicago, IL) and JMP 9.0 for Windows. The genotypes and allele frequencies associated with the susceptibility of KD and disease outcomes (CAL formation, IVIG treatment response, and aneurysm) were analyzed with the *χ*^2^ test. The *χ*^2^ test with 1 degree of freedom was used to perform the Hardy-Weinberg equilibrium. Linkage disequilibrium (LD) was assessed for haplotype blocks, which were defined using the default setting of the Haploview software 4.1. Two-sided *P* values <0.05 were considered statistically significant.

## RESULTS

### Association of *IFNG* Gene Polymorphisms With Susceptibility to KD

**FIGURE 1:**
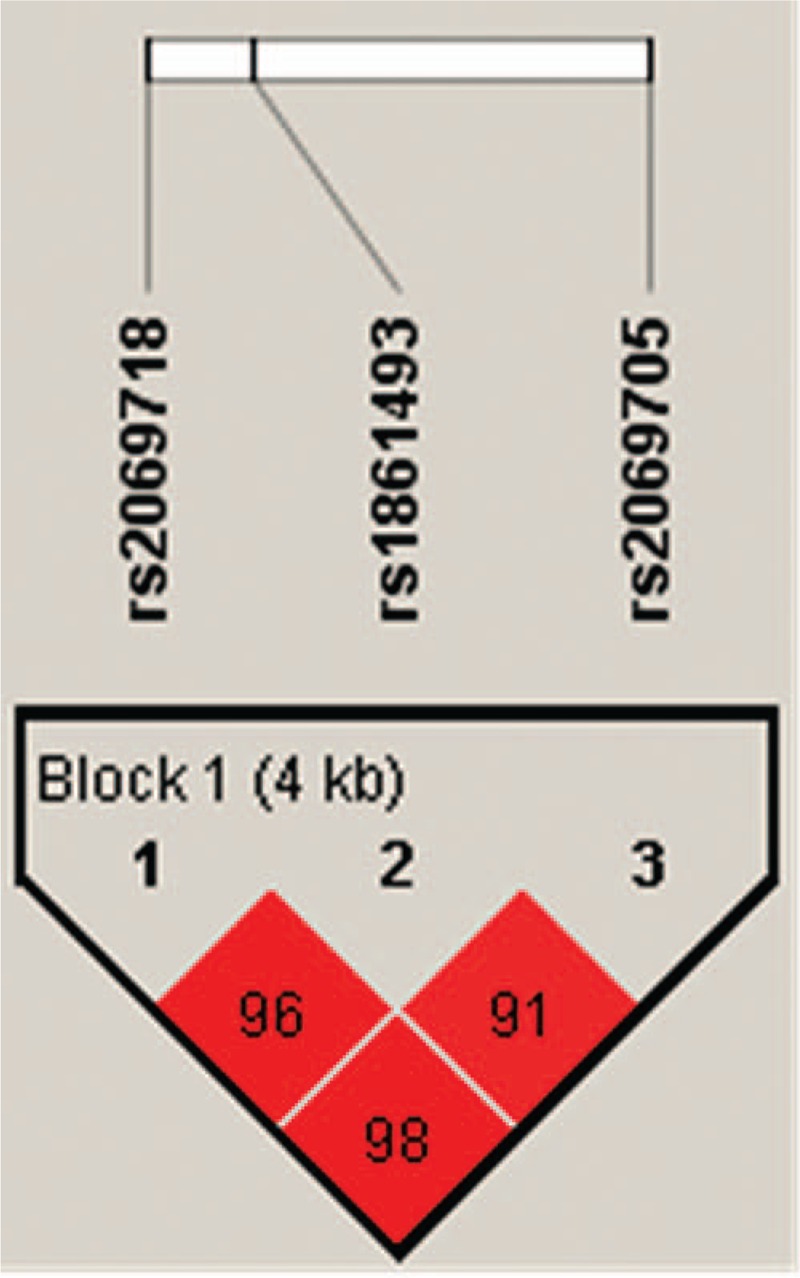
Linkage disequilibrium plot of *IFNG* gene and the haplotype block structure of rs2069718, rs1861493, and rs2069705 in Kawasaki disease patients. The number on the cell is the logarithm of the odds score of D’.

Table 1 demonstrates the clinical background in this study. There were 381 KD patients and 569 controls in this study. Male subjects accounted for 66.8% of patients of KD and 56% of controls. The mean ages of the cases and controls were 1.7 ± 1.6 years and 5.7 ± 4.9 years, respectively, with children predominantly in this study. In total, 12.9% of patients of KD were resistant to initial IVIG treatment, 9.7% development with CAL, and 4.2% with CAA. The SNP genotypes were in line with the Hardy-Weinberg equilibrium for cases and controls (Table 2). Figure 1 showed the LD plot of IFN-γ gene and the haplotype block structure of rs2069718, rs1861493, and rs2069705 in KD patients. In the comparison of the allele distribution between the KD patients and controls, rs2069718 of *IFNG* showed significant associations with KD under the genotype model (*P* = 0.0242).

**TABLE 1:**
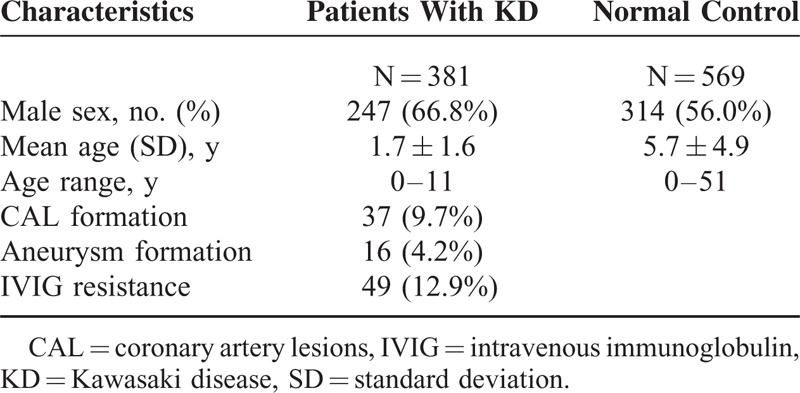
Basal Characteristics of KD Patients and Normal Controls

**TABLE 2:**
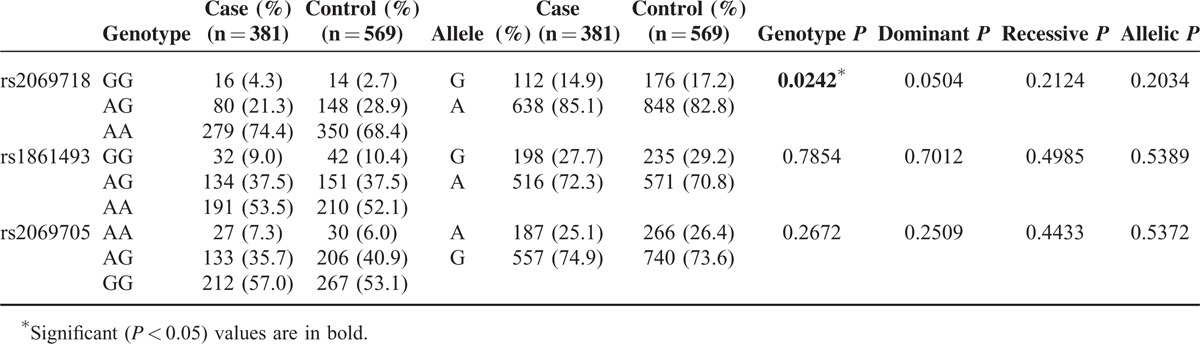
Genotype and Allele Frequencies of the *IFNG* Gene in Controls and Patients With Kawasaki Disease

### Association Between *IFNG* Gene Polymorphisms and Aneurysm Formation and IVIG Responsiveness

As shown in Table 3, the G allele of rs1861493 conferred a better response to IVIG treatment in KD patients (*P* = 0.0407). In addition, the dominant model of G allele frequency of rs1861493 conferred a significantly lower risk for CAA in KD patients (*P* = 0.0250) (Table 4). However, in the comparison of the allele distribution and the risk of CAL formation, there were no differences between the genotypes of *IFNG* and CAL formation (*P* = 0.2924 in rs2069718, *P* = 0.6057 in rs1861493, *P* = 0.2456 in rs2069705).

**TABLE 3:**
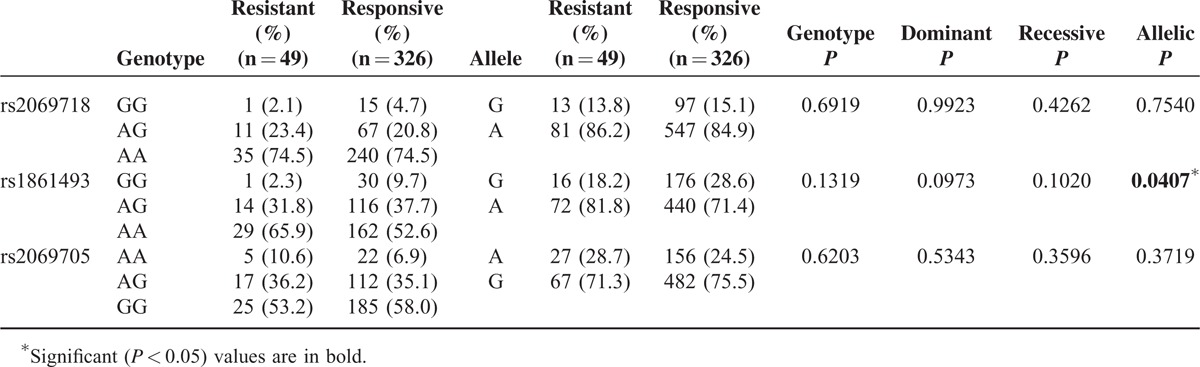
Genotyping and Allele Frequency of *IFNG* Single-Nucleotide Polymorphisms in Patients With Kawasaki Disease Responding or Not Responding to Intravenous Immunoglobulin Treatment

**TABLE 4:**
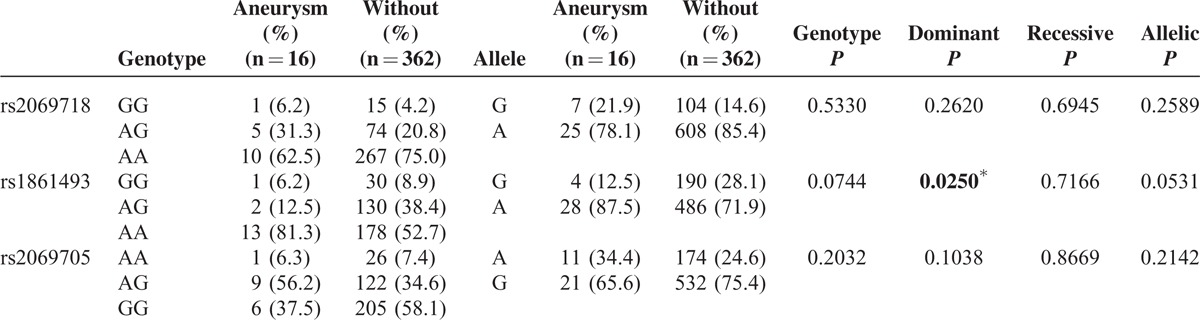
Genotyping and Allele Frequency of *IFNG* Single-Nucleotide Polymorphism in Patients With Kawasaki Disease With Aneurysm or Without Aneurysm

### Plasma IFN-γ Levels in KD Patients With different *IFNG* Genotypes

Then, we investigated the association between *IFNG* genetic variation and the plasma IFN-γ levels in KD patients. As shown in Tables 5 and 6, there was a significant genetic difference between rs1861493 of *IFNG* and the plasma IFN-γ levels in KD patients. The plasma IFN-γ levels were lower in the AA allele than in the GG allele of rs1861493 both before and after IVIG treatment (*P* = 0.008 and 0.009, respectively). However, there was no genetic variation association between rs2069718 and rs2069705 of *IFNG* and the plasma IFN-γ levels in KD patients.

**TABLE 5:**
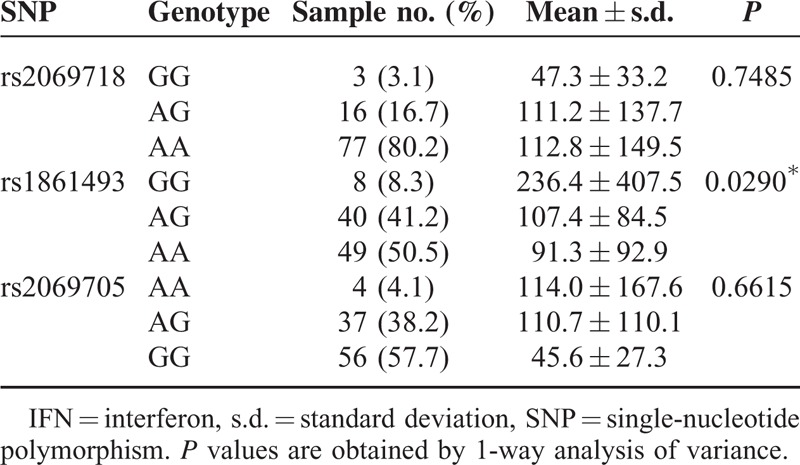
Associations of Plasma IFN-γ With Genotype in Kawasaki Disease Patients Before Intravenous Immunoglobulin

**TABLE 6:**
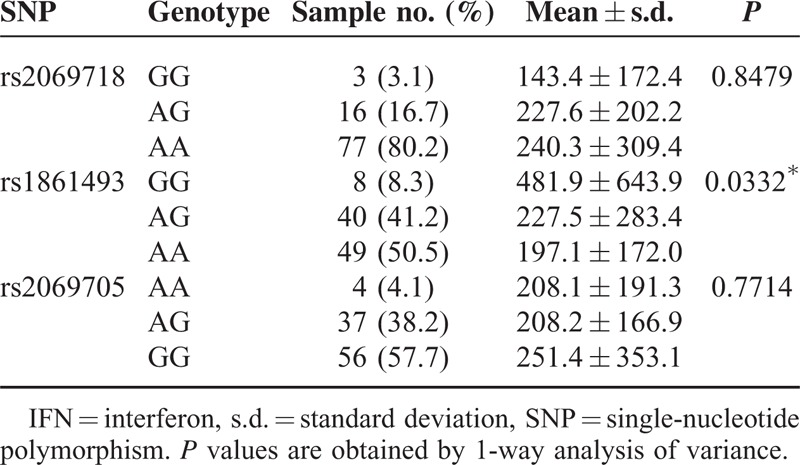
Associations of Plasma IFN-γ With Genotype in Kawasaki Disease Patients After Intravenous Immunoglobulin Treatment

## DISCUSSION

The pathogenesis of KD remains unknown and KD results from an undefined infectious process in a genetically predisposed individual with a “double hit model.”^25^ Several studies have indicated an imbalanced immunity between Th1/Th2 response in KD patients.^26^ In this study, we found that there was an *IFNG* genotype difference between the normal controls and KD patients. The G allele of rs1861493 conferred a better response to IVIG treatment in KD patients. In addition, the AA allele frequencies of rs1861493 were associated with a significantly higher risk of CAA in KD patients. Furthermore, the plasma IFN-γ levels were lower in the AA allele than in the GG allele of rs1861493 both before and after IVIG treatment in KD patients.

Although growing evidence indicates that the cytokine profiles are associated with the pathogenesis of KD, the precise immunopathogenesis of KD remains unclear. Recently, we have shown that Th1/Th2^6^ and Th17/Treg^27^ cytokine profiles are associated with the pathogenesis of KD, and this significantly predicted the treatment and prognosis of KD.^7^ Recently, Wang et al^12^ have demonstrated that the IFN-γ levels in the main KD patients appeared to stay within the normal range, and that was consistent with Matsubara et al's^28^ observation that only 30% of KD patients show a slightly increased IFN-γ level. They also found a decrease in IFN-γ-producing CD3+ T cells during the acute stage of KD.^29^ Although no previous report has linked IFN-γ levels with risk of CAA in the KD patients, lower IFN-γ levels are associated with abdominal aortic aneurysm^30,31^ and popliteal artery aneurysms formation.^32^ Moreover, by using expression quantitative trait locus meta-analysis, the genotype of rs1861493 showed a significant association with *IFNG* gene expression in blood,^33^ which provides a reliable explanation of the association of rs1861493 and the IFN-γ level. Supporting this observation, our study has found that IFN-γ levels were lower in the AA allele of rs1861493, which was associated with a significantly higher risk of CAA in KD patients.

*IFNG* gene polymorphisms have also been involved in asthma susceptibility,^13,14,34^ infectious disease,^15^ coronary artery disease,^16^ autoimmune diseases,^17,18,35^ and type I diabetes mellitus^36^ in some genetic association studies. Kumar et al^13^ first described a significant association of an intronic SNP of the *IFNG* gene with asthma and identified the association of rs1861494 A/G polymorphism with asthma, which may control the IFN-γ levels and thus modulate asthma pathogenesis. In accordance with these findings, we also observed an expression difference between the AA allele and GG allele in KD patients. Moreover, the rs1861494 has been associated with white adults with polymyositis,^37^ risk of development of pulmonary tuberculosis,^38^ and the symptomatic score of acute Q fever.^39^

Growing evidence suggests that genetics play a major role in the immunopathogenesis of KD^26^ and findings in the past decade have contributed to a major breakthrough in the genetic study of KD. In Asia, Onouchi et al^40^ discovered a susceptibility loci on chromosome 19q13.2, and further identified the functional SNP of inositol 1,4,5-trisphosphate 3-kinase C in the disease activity of KD. Furthermore, the identification of several genomic regions has been linked to the pathogenesis of KD, including CD40,^41^ BLK,^41^ TARC/CCL17,^7^ and FCGR2A.^42^ In addition, we also observed an association between genomic hypomethylation of FCGR2A and susceptibility to KD and IVIG resistance.^43^ In this study, we revealed that *IFNG* gene polymorphisms were not only associated with the susceptibility of KD, but were also significantly associated with disease outcomes of KD patients of Taiwanese population.^43^ Therefore, future studies of *IFNG* gene polymorphisms in KD patients of other ethnic groups could be interesting and important.

In conclusion, our results gain insights into the association of *IFNG* gene polymorphisms with the susceptibility of KD as well as *IFNG* gene polymorphisms and the plasma IFN-γ levels with the development of CAA in KD patients.
